# *Child and Adolescent Psychiatry and Mental Health* reviewer acknowledgement 2014

**DOI:** 10.1186/s13034-015-0034-y

**Published:** 2015-02-06

**Authors:** Jörg M Fegert

**Affiliations:** Department of Child and Adolescent Psychiatry and Psychotherapy, University of Ulm, Steinhoevelstr. 5, Ulm, 89075 Germany

## Abstract

The editors of *Child and Adolescent Psychiatry and Mental Health* would like to thank all of our reviewers who have contributed to the journal in volume 8 (2014).

**Marc Allroggen**

Germany

**Martijn Arns**

Netherlands

**Josephine Barbaro**

Australia

**Claus Barkmann**

Greenland

**Sheri Bauman**

United States of America

**Michele Baumann**

Luxembourg

**Myron Belfer**

United States of America

**Vashti Berry**

United Kingdom

**Maria Teresa Botello**-**Harbaum**

United States of America

**David Buchbinder**

United States of America

**Larry Burd**

United States of America

**Marcy Burstein**

United States of America

**Franco Cavallo**

Italy

**David Chambers**

United States of America

**Yiong Huak Chan**

Singapore

**Deena Chisolm**

United States of America

**Joanna Ting Wai Chu**

New Zealand

**Antonio Clavenna**

Italy

**Paula Cloutier**

Canada

**Melissa Cortina**

United Kingdom

**Georgina Cox**

Australia

**Michelle Dawson**

Canada

**Rafael de Assis da Silva**

Brazil

**Arden Dingle**

United States of America

**Joseph Durlak**

United States of America

**Pia Enebrink**

Sweden

**Elif Ercan**

Turkey

**Philip Fisher**

United States of America

**Carol Fitzpatrick**

Ireland

**Bacy Fleitlich**-**Bilyk**

Brazil

**Lila Fleming**

United States of America

**Marjolein Fokkema**

Netherlands

**Daniel Fung**

Singapore

**Holger Gevensleben**

Germany

**Laurence Greenhill**

United States of America

**Johannes Haerting**

Germany

**Charlotte Hanisch**

Germany

**Lisa Harryson**

Sweden

**Nina Heinrichs**

Germany

**Pieter J Hoekstra**

Netherlands

**Embry Howell**

United States of America

**Tina In**-**Albon**

Germany

**Sigrid James**

United States of America

**Bruce Jonas**

United States of America

**Jennifer Jordan**

New Zealand

**Niina Junttila**

Finland

**Jon Jureidini**

Australia

**Espérance Kashala**

Congo Democratic Republic

**Ferdinand Keller**

Germany

**David Kemp**

United States of America

**Bonnie Kerker**

United States of America

**Suzanne Kerns**

United States of America

**Praveen Khairkar**

India

**Christian Kieling**

Brazil

**Hyun**-**Sil Kim**

South Korea

**Bryan King**

United States of America

**Michael Kohn**

Australia

**Anne Katrin Künster**

Germany

**Natasha Latzman**

United States of America

**Elizabeth Letourneau**

United States of America

**Ching**-**Hua Lin**

Taiwan

**Yu**-**Ju Lin**

Taiwan

**Arnold Lohaus**

Germany

**Astri J. Lundervold**

Norway

**Aristides Machado**-**Rodrigues**

Portugal

**Stefania Maggi**

Canada

**Rajneesh Mahajan**

United States of America

**Andres Martin**

United States of America

**Therese Mathews**

United States of America

**Jon McClellan**

United States of America

**Christopher McDougle**

United States of America

**Joe McNamara**

United States of America

**Anilena Mejia**

United Kingdom

**Susannah Melville**

United Kingdom

**Eugen Mengel**

Germany

**Inmaculada Moreno García**

Spain

**Tally Moses**

United States of America

**Stella Muthuri**

Canada

**Kerstin Neander**

Sweden

**Dennis Nitkowski**

Germany

**Ingrid Landgraff Oestlie**

Norway

**Thomas Olino**

United States of America

**Susanna Payne**

United Kingdom

**Geraldine Pearson**

United States of America

**Wendy Plante**

United States of America

**Paul L. Plener**

Germany

**Guilherme Polanczyk**

Brazil

**Maria Cristina Porfirio**

Italy

**Irina Elizabeth Poslawsky**

Netherlands

**Jonathan Prince**

United States of America

**Daniel Radeloff**

Germany

**Nicola Reavley**

Australia

**Ruth Reed**

United Kingdom

**Nicole Reilly**

Australia

**Matthias Reitzle**

Germany

**Thiago Rocha**

Brazil

**Paul Rohde**

United States of America

**Jeanne Ryan**

United States of America

**Per**-**Anders Rydelius**

Sweden

**Christina Sakai**

United States of America

**Giovanni Salum**

Brazil

**Masafumi Sanefuji**

Japan

**Ursula Sansom**-**Daly**

Australia

**Caferi Tayyar Sasmaz**

Turkey

**Susan Sawyer**

Australia

**Erin Schoenfelder**

United States of America

**Thomas Schofield**

United States of America

**Fiona Schulte**

Canada

**Donald Sharpe**

Canada

**Jordan Sibeoni**

France

**Inga Dora Sigfusdottir**

Iceland

**Ilina Singh**

United Kingdom

**Petros Skapinakis**

Greece

**Tristram Smith**

United States of America

**Gottfried Spangler**

Germany

**Anthony Spirito**

United States of America

**Fabio Sticca**

Switzerland

**Lisanne Stone**

Netherlands

**Carla Stover**

United States of America

**James Swanson**

United States of America

**Katalin Szakszon**

Hungary

**Toshinobu Takeda**

Japan

**Sarah Tarbox**

United States of America

**Cristiano Tschiedel Belem da Silva**

Brazil

**Aina Basilier Vaage**

Norway

**Mitch van Geel**

Netherlands

**Benedetto Vitiello**

United States of America

**Jan Wallander**

United States of America

**James Waxmonsky**

United States of America

**Carl Weems**

United States of America

**Yifeng Wei**

Canada

**Gunda Wößner**

Germany

**Peter Zimmermann**

Germany

